# Supplementary material for: Spin-phonon relaxation from a universal ab initio density-matrix approach
Source: Nat Commun. 2020 Jun 3;11:2780. doi: 10.1038/s41467-020-16063-5 (PMC7270186; doi:10.1038/s41467-020-16063-5)
Supplement: Supplementary file 1 — Supplementary Information [file 41467_2020_16063_MOESM1_ESM.pdf]

Supplementary information for: Spin-phonon relaxation in disparate materials from a universal *ab initio* density matrix approach

Junqing Xu‡,[1] Adela Habib‡,[2] Sushant Kumar,[2] Feng Wu,[1] Ravishankar Sundararaman†,[2] and Yuan Ping*[1]

[1]*Department of Chemistry and Biochemistry, University of California, Santa Cruz, CA 95064, USA*
[2]*Department of Materials Science and Engineering, Rensselaer Polytechnic Institute, 110 8th Street, Troy, New York 12180, USA*

(Dated: April 6, 2020)

Supplementary Note 1: Formalism of spin relaxation time

Starting from the Lindblad master equation of density matrix in interaction picture based on the standard Born-Markov approximation given in Ref. 1

$$
\begin{aligned}
\frac{\partial \rho_{\alpha_1\alpha_2}}{\partial t} =& \frac{2\pi}{\hbar N_q}\mathrm{Re}\sum_{q\lambda\pm\alpha'\alpha_1'\alpha_2'}\left[\begin{array}{c}(I-\rho)_{\alpha_1\alpha'}\left(G^{q\lambda\pm}\right)_{\alpha'\alpha_1'}\rho_{\alpha_1'\alpha_2'}\left(G^{q\lambda\mp}\right)_{\alpha_2'\alpha_2}\\ -\left(G^{q\lambda\mp}\right)_{\alpha_1\alpha'}(I-\rho)_{\alpha'\alpha_1'}\left(G^{q\lambda\pm}\right)_{\alpha_1'\alpha_2'}\rho_{\alpha_2'\alpha_2}\end{array}\right]n_{q\lambda}^{\pm},\\
G_{kk'}^{q\lambda\mp} =& g_{kk'}^{\lambda\mp}\sqrt{\delta\left(\epsilon_k-\epsilon_{k'}\mp\omega_{q\lambda}\right)},\\
g_{kk'}^{q\lambda\mp} =& \sum_{R,i}\sqrt{\frac{\hbar}{2M_R\omega_{q\lambda}}}<k|\frac{\delta V^{\mathrm{KS}}}{\delta R_i}\cdot e_{q\lambda}(R,i)\,|k'>\delta_{q\mp(k-k')},
\end{aligned}
\tag{1}
$$

where α is the combined index of electron wavevector k and band index n, q is phonon wavevector, λ is normal mode, $n_{q\lambda}^{\pm}=n_{q\lambda}+0.5\pm0.5$ and $n_{q\lambda}$ is the phonon occupation number, R is the atomic coordinate, $i=x,y,z$, $e_{q\lambda}(R,i)$ is the phonon polarization vector and $\delta\left(\epsilon_k-\epsilon_{k'}\mp\omega_{q\lambda}\right)$ is the Dirac delta function.

In its matrix form, the above equation becomes

$$
\frac{\partial\rho}{\partial t}=\frac{2\pi}{\hbar N_q}\mathrm{Re}\sum_{q\lambda\pm}\left[\,(I-\rho)\,G^{q\lambda\pm}\rho G^{q\lambda\mp}-G^{q\lambda\mp}(I-\rho)\,G^{q\lambda\pm}\rho\,\right]n_{q\lambda}^{\pm}. \tag{2}
$$

Suppose the density matrix $\rho=\rho^{\mathrm{eq}}+\delta\rho$, where ρ^{eq} is the density matrix of the final equilibrium state and $||\delta\rho||\ll||\rho^{\mathrm{eq}}||$.

$$
\frac{\partial\rho}{\partial t}=\frac{2\pi}{\hbar N_q}\mathrm{Re}\sum_{q\lambda\pm}\left[\begin{array}{c}(I-\rho^{\mathrm{eq}}-\delta\rho)\,G^{q\lambda\pm}(\rho^{\mathrm{eq}}+\delta\rho)\,G^{q\lambda\mp}\\ -G^{q\lambda\mp}(I-\rho^{\mathrm{eq}}-\delta\rho)\,G^{q\lambda\pm}(\rho^{\mathrm{eq}}+\delta\rho)\end{array}\right]n_{q\lambda}^{\pm}. \tag{3}
$$

Considering $\frac{\partial\rho}{\partial t}|_{\rho=\rho^{\mathrm{eq}}}\equiv0$, ρ^{eq} is just Fermi-dirac distribution function f and neglecting second-order terms, we obtain

$$
\frac{\partial\rho}{\partial t}=\frac{2\pi}{\hbar N_q}\mathrm{Re}\sum_{q\lambda\pm}\left[\begin{array}{c}-\delta\rho G^{q\lambda\pm}fG^{q\lambda\mp}+(I-f)\,G^{q\lambda\pm}\delta\rho G^{q\lambda\mp}\\ +G^{q\lambda\mp}\delta\rho G^{q\lambda\pm}f-G^{q\lambda\mp}(I-f)\,G^{q\lambda\pm}\delta\rho\end{array}\right]n_{q\lambda}^{\pm}. \tag{4}
$$

Given an exponentially-relaxing measured quantity $O=\mathrm{Tr}(o\rho)$, where o is the observable operator, we can define the relaxation rate Γ_o and relaxation time $\tau_o=\Gamma_o^{-1}$ of quantity O as

---

‡ JX and AH contributed equally to this work.
† sundar@rpi.edu
* yuanping@ucsc.edu

$$\frac{\partial \left(O-O^{\rm eq}\right)}{\partial t}=-\,\Gamma_o(O-O^{\rm eq}),\tag{5}$$

where

$$\Gamma_o=-\,\frac{2\pi}{\hbar N_q {\rm Tr}\left(o\delta\rho\right)}{\rm TrRe}\sum_{q\lambda\pm}\left\{\begin{array}{c}\left[-\delta\rho G^{q\lambda\pm}f+\left(I-f\right)G^{q\lambda\pm}\delta\rho\right]G^{q\lambda\mp}o\\ +oG^{q\lambda\mp}\left[\delta\rho G^{q\lambda\pm}f-\left(I-f\right)G^{q\lambda\pm}\delta\rho\right]\end{array}\right\}n_{q\lambda}^{\pm},\tag{6}$$

Then,

$$\begin{aligned}\Gamma_o=&-\,\frac{2\pi}{\hbar N_q {\rm Tr}\left(o\delta\rho\right)}{\rm TrRe}\sum_{q\lambda}\left\{\begin{array}{c}\left[o,G^{q\lambda-}\right]\left[\delta\rho G^{q\lambda+}fn_{q\lambda}^{+}-\left(I-f\right)n_{q\lambda}^{+}G^{q\lambda+}\delta\rho\right]\\ +\left[o,G^{q\lambda+}\right]\left[\delta\rho G^{q\lambda-}fn_{q\lambda}^{-}-\left(I-f\right)n_{q\lambda}^{-}G^{q\lambda-}\delta\rho\right]\end{array}\right\}\\ =&\frac{2\pi}{\hbar N_q {\rm Tr}\left(o\delta\rho\right)}{\rm TrRe}\sum_{q\lambda}\left\{\begin{array}{c}\left[o,G^{q\lambda-}\right]\left[\delta\rho G^{q\lambda-}\left(I-f\right)n_{q\lambda}^{+}-fn_{q\lambda}^{+}G^{q\lambda-}\delta\rho\right]^{\dagger}\\ +\left[o,G^{q\lambda-}\right]^{\dagger}\left[\delta\rho G^{q\lambda-}fn_{q\lambda}^{-}-\left(I-f\right)n_{q\lambda}^{-}G^{q\lambda-}\delta\rho\right]\end{array}\right\}.\end{aligned}\tag{7}$$

Taking complex conjugate of the second terms, we have

$$\Gamma_o=\frac{2\pi}{\hbar N_q {\rm Tr}\left(o\delta\rho\right)}{\rm TrRe}\sum_{q\lambda}\left[o,G^{q\lambda-}\right]\left[\delta\rho G^{q\lambda-}\left(n_{q\lambda}+I-f\right)-\left(n_{q\lambda}+f\right)G^{q\lambda-}\delta\rho\right]^{\dagger},\tag{8}$$

where $\left[o,G^{q\lambda-}\right]$ is a commutator of two matrices. Assuming $\delta\rho$ and o are both k-diagonal, which are usually true,

$$\Gamma_o=\frac{2\pi}{\hbar N_q Tr\left(o\delta\rho\right)}{\rm Tr}_n{\rm Re}\sum_{kk'\lambda}\left[o,G^{q\lambda-}\right]_{kk'}\left[\begin{array}{c}\left(\delta\rho\right)_k G_{kk'}^{q\lambda-}\left(n_{q\lambda}+I-f_{k'}\right)\\ -\left(n_{q\lambda}+f_k\right)G_{kk'}^{q\lambda-}\left(\delta\rho\right)_{k'}\end{array}\right]^{\dagger_n},\tag{9}$$

where Tr_n and $\dagger_n$ are trace and matrix complex conjugate for only band index.

Supplementary Note 2: Fermi's-golden-rule-like formula

The most general (experiment-agnostic) choice of $\delta\rho$ for preparing a spin polarization is to assume that all other degrees of freedom are in thermal equilibrium, which can be implemented using a test magnetic field B_i as a Lagrange multiplier for implementing a spin polarization constraint. Suppose a perturbation $H_1=-2\mu_{\rm B}B_iS_i/\hbar$ is turned on at $t\rightarrow-\infty$ but is turned off at $t=0$. After that, the spin starts to relax, finally at $t\rightarrow+\infty$, the system goes back to the final equilibrium state. At $t=0^-$, the system is still at a equilibrium state with Hamiltonian $H=H_0+H_1$. Let V_1 be the eigenvectors of this equilibrium state at first order of perturbation theory. With V_1, the density matrix at $t=0^-$ is the Fermi function with eigenvalues $\epsilon+\epsilon_1$. With ground-state eigenvectors, $\rho\left(t=0^-\right)=V_1f\left(\epsilon+\epsilon_1\right)V_1^{\dagger}$.

Following the instruction of degenerate perturbation theory, for $H=H_0+H_1$, we firstly define the degenerate-subspace projection of H_1, $H_{1,nn'}^{\rm deg}\equiv H_{1,nn'}\delta_{\epsilon_n\epsilon_{n'}}$, and diagonalize it $H_1^{\rm deg}=V_1^{\rm deg}\Lambda_1^{\rm deg}\left(V_1^{\rm deg}\right)^{\dagger}$. The diagonal elements of $\Lambda_1^{\rm deg}$ are just ϵ_1 under first-order perturbation. Next, apply the non-degenerate perturbation theory to eigenvectors,

$$\begin{aligned}V_1=&V_1^{\rm deg}\left(I-A\right),\\ A_{mn}=&\frac{\left\langle V_1^{\rm deg}\right|H_1\left|V_1^{\rm deg}\right\rangle_{mn}}{\epsilon_m-\epsilon_n}\left(1-\delta_{\epsilon_m,\epsilon_n}\right).\end{aligned}\tag{10}$$

Taking the zeroth and first order of $\rho\left(t=0^-\right)$,

$$\begin{aligned}\rho\left(t=0^{-}\right)=&\left|V_1\right\rangle f\left(\epsilon+\epsilon_1\right)\left\langle V_1\right|\\=&\left|V_1^{\deg}\right\rangle\left\langle V_1^{\deg}|V_1\right\rangle f\left(\epsilon+\epsilon_1\right)\left\langle V_1|V_1^{\deg}\right\rangle\left\langle V_1^{\deg}\right|\\=&\left|V_1^{\deg}\right\rangle\left(I-A\right)f\left(\epsilon+\epsilon_1\right)\left(I-A^{\dagger}\right)\left\langle V_1^{\deg}\right|\\\approx&\left|V_1^{\deg}\right\rangle\left(f\left(\epsilon+\epsilon_1\right)-Af\left(\epsilon+\epsilon_1\right)-f\left(\epsilon+\epsilon_1\right)A^{\dagger}\right)\left\langle V_1^{\deg}\right|.\end{aligned}\tag{11}$$

Notice that $A^{\dagger}=-A$, we have

$$\rho\left(t=0^{-}\right)=\left|V_1^{\deg}\right\rangle\left(f\left(\epsilon+\epsilon_1\right)+f\left(\epsilon+\epsilon_1\right)A-Af\left(\epsilon+\epsilon_1\right)\right)\left\langle V_1^{\deg}\right|.\tag{12}$$

Therefore, with the basis $V_1^{\deg}$, $\delta\rho=\rho\left(t=0^{-}\right)-\rho^{\mathrm{eq}}$, where $\rho_{eq}=f(\epsilon)$ is the equilibrium density matrix, is

$$\begin{aligned}\left\langle V_1^{\deg}\right|\delta\rho\left|V_1^{\deg}\right\rangle_{mn}=&\left[f\left(\epsilon_m+\Lambda_{1,mm}\right)-f\left(\epsilon_m\right)\right]\delta_{\epsilon_m,\epsilon_n}\\&+\frac{f\left(\epsilon_m+\Lambda_{1,mm}\right)-f\left(\epsilon_n+\Lambda_{1,nn}\right)}{\epsilon_m-\epsilon_n}\left\langle V_1^{\deg}\right|H_1\left|V_1^{\deg}\right\rangle_{mn}\left(1-\delta_{\epsilon_m,\epsilon_n}\right)\\\approx&\frac{\partial f}{\partial\epsilon}|_{\epsilon=\epsilon_m}\Lambda_{1,mm}\delta_{\epsilon_m,\epsilon_n}+\frac{f\left(\epsilon_m\right)-f\left(\epsilon_n\right)}{\epsilon_m-\epsilon_n}\left\langle V_1^{\deg}\right|H_1\left|V_1^{\deg}\right\rangle_{mn}\left(1-\delta_{\epsilon_m,\epsilon_n}\right).\end{aligned}\tag{13}$$

Considering that $\frac{\partial f}{\partial\epsilon}|_{\epsilon=\epsilon_m}=\lim_{\epsilon_m\to\epsilon_n}\frac{f(\epsilon_m)-f(\epsilon_n)}{\epsilon_m-\epsilon_n}$, we can write

$$\left\langle V_1^{\deg}\right|\delta\rho\left|V_1^{\deg}\right\rangle_{mn}=\frac{f\left(\epsilon_m\right)-f\left(\epsilon_n\right)}{\epsilon_m-\epsilon_n}\left\langle V_1^{\deg}\right|H_1\left|V_1^{\deg}\right\rangle_{mn}.\tag{14}$$

Obviously, with any set of eigenstates of H_0, which is a unitary transform of $V_1^{\deg}$, we have

$$\delta\rho_{mn}=\frac{f\left(\epsilon_m\right)-f\left(\epsilon_n\right)}{\epsilon_m-\epsilon_n}H_{1,mn}=-\frac{2\mu_{\mathrm{B}}B_i}{\hbar}\frac{f\left(\epsilon_m\right)-f\left(\epsilon_n\right)}{\epsilon_m-\epsilon_n}S_{i,mn}.\tag{15}$$

For the systems we studied in this work, we notice that $S_{i,k,mn}\approx0$ when $\epsilon_{km}\neq\epsilon_{kn}$. Therefore, we have

$$\delta\rho\approx-\frac{2\mu_{\mathrm{B}}B_i}{\hbar}\frac{\partial f}{\partial\epsilon}S_i^{\deg},\tag{16}$$

where $(S_i^{\deg})_{knn'}\equiv(S_i)_{knn'}\delta_{\varepsilon_{kn}\varepsilon_{kn'}}$ is the degenerate-subspace projection of S_i.
Insert the above equations in Eq. 9,

$$\Gamma_{s,i}=\frac{2\pi}{\hbar N_q\mathrm{Tr}\left(S_i\delta\rho\right)}\mathrm{Tr}_n\mathrm{Re}\sum_{kk'\lambda}\left[S_i,G^{q\lambda-}\right]_{kk'}\left[\begin{array}{c}\left(-f_k'\right)S_{i,k}^{\deg}G_{kk'}^{q\lambda-}\left(n_{q\lambda}+I-f_{k'}\right)\\-\left(n_{q\lambda}+f_k\right)G_{kk'}^{q\lambda-}\left(-f_{k'}'\right)S_{i,k'}^{\deg}\end{array}\right]^{\dagger_n}.\tag{17}$$

When delta functions in $G_{kk'}^{q\lambda-}$ is exact, i.e., $\epsilon_k=\epsilon_{k'}+\omega_{q\lambda}$, we have the following relations

$$\begin{aligned}n_{q\lambda}=&\frac{f_k\left(1-f_{k'}\right)}{f_{k'}-f_k}\\n_{q\lambda}+f_k=&\frac{f_k\left(1-f_k\right)}{f_{k'}-f_k},\\n_{q\lambda}+1-f_{k'}=&\frac{f_{k'}\left(1-f_{k'}\right)}{f_{k'}-f_k}.\end{aligned}\tag{18}$$

Therefore,

$$\Gamma_{s,i} = \frac{2\pi}{\hbar N_q \mathrm{Tr}\left[S_i\left(\partial f/\partial\epsilon\right)S_i^{\mathrm{deg}}\right]k_{\mathrm{B}}T}\mathrm{Tr}_n\mathrm{Re}\sum_{kk'\lambda}\left[S_i, G^{q\lambda-}\right]_{kk'} f_{k'}\left(\left[S_i^{\mathrm{deg}}, G^{q\lambda-}\right]_{kk'}\right)^{\dagger_n}\left(1-f_k\right)n_{q\lambda}. \tag{19}$$

Utilizing further the exact energy-conserving conditions implied by delta functions of both $G^{q\lambda-}$ (defined in Eq.1) in two commutators, we will have

$$\begin{aligned}\Gamma_{s,i} =& \frac{2\pi}{\hbar N_q \mathrm{Tr}\left[\left(\partial f/\partial\epsilon\right)|S_i^{\mathrm{deg}}|^2\right]k_{\mathrm{B}}T}\mathrm{Tr}_n\mathrm{Re}\sum_{kk'\lambda}\left[S_i^{\mathrm{deg}}, G^{q\lambda-}\right]_{kk'} f_{k'}\left(\left[S_j^{\mathrm{deg}}, G^{q\lambda-}\right]_{kk'}\right)^{\dagger_n}\left(1-f_k\right)n_{q\lambda}\\ =& \frac{2\pi}{\hbar N_q \mathrm{Tr}\left[\left(\partial f/\partial\epsilon\right)|S_i^{\mathrm{deg}}|^2\right]k_{\mathrm{B}}T}\mathrm{Tr}_n\mathrm{Re}\sum_{knk'n'\lambda}\left|\left[S_i^{\mathrm{deg}}, g^{q\lambda-}\right]_{knk'n'}\right|^2\delta\left(\epsilon_{kn}-\epsilon_{k'n'}-\omega_{q\lambda}\right)f_{k'n'}\left(1-f_{kn}\right)n_{q\lambda}.\end{aligned} \tag{20}$$

Such formula is preferred numerically since $\Gamma_{s,i}$ is positively defined.

For two Kramers degenerate bands with weak spin mixing, $g_{kk'}^{q\lambda-}$ has the form $\begin{pmatrix} g_{kk'}^{\lambda,\Uparrow\Uparrow} & g_{kk'}^{\lambda,\Uparrow\Downarrow} \\ g_{kk'}^{\lambda,\Uparrow\Downarrow,*} & g_{kk'}^{\lambda,\Uparrow\Uparrow}\end{pmatrix}$. After diagonalization, the spin matrices $S_{z,k} \equiv S_{z,k}^{\mathrm{deg}}$ will have diagonal elements $\pm\left(1-b_k^2\right)$, where $b_k^2 \ll 1$ is the spin mixing parameter. Therefore,

$$\begin{aligned}\left[S_i^{\mathrm{deg}}, g^{q\lambda-}\right]_{kk'} &= \begin{bmatrix}\left(b_{k'}^2-b_k^2\right)g_{kk'}^{\lambda,\Uparrow\Uparrow} & \left(2-b_k^2-b_{k'}^2\right)g_{kk'}^{\lambda,\Uparrow\Downarrow}\\ \left(2-b_k^2-b_{k'}^2\right)g_{kk'}^{\lambda,\Uparrow\Downarrow,*} & \left(b_k^2-b_{k'}^2\right)g_{kk'}^{\lambda,\Uparrow\Uparrow}\end{bmatrix}\\ &\approx \begin{bmatrix} 0 & 2g_{kk'}^{\lambda,\Uparrow\Downarrow}\\ 2g_{kk'}^{\lambda,\Uparrow\Downarrow,*} & 0\end{bmatrix},\end{aligned} \tag{21}$$

and

$$\Gamma_{s,i} = \frac{8\pi}{\hbar N_q \partial f_k/\partial\epsilon k_{\mathrm{B}}T}\mathrm{Tr}_n\mathrm{Re}\sum_{kk'\lambda}\left|g_{kk'}^{\lambda,\Uparrow\Downarrow}\right|^2\delta\left(\epsilon_{kn}-\epsilon_{k'n'}-\omega_{k-k',\lambda}\right)f_{k'n'}\left(1-f_{kn}\right)n_{k-k',\lambda}. \tag{22}$$

The above formula is the same as Eq. 3 and 4 in Ref. 2.

Supplementary Note 3: Wannier-interpolated band structure

Supplementary Figure 1-3 show that our Wannier-interpolated band structures fit perfectly those directly calculated by density functional theory (DFT). This ensures the high quality of the Wannierization, which is crucial to obtain accurate lifetime results.

Supplementary Note 4: Phonon dispersion

Supplementary Figure 4 shows the phonon band structure of monolayer MoS_2 and $MoSe_2$. The lowest three modes are the out-of-plane acoustic or flexural (ZA), transverse acoustic (TA) and longitudinal acoustic (LA) modes. Both the TA and LA modes show linear dispersion in the long wavelength limit, while the ZA phonons deviate with an approximate quadratic dependence. The next two are in-plane optical E" modes, with two S(Se) atoms vibrating out of phase and the Mo atomic static. The TO and polar LO modes are two in-plane modes, but with all three atoms moving out of phase. The LO-TO splitting of MoS_2 is is too small to be visible. The A1 and A2" phonons are out-of-plane optical vibrations. More specifically, the A1 mode has the Mo atom static while the S(Se) atoms moving in the opposite directions. All three atoms oscillate out of the plane and out of phase in the case of A2" phonons.

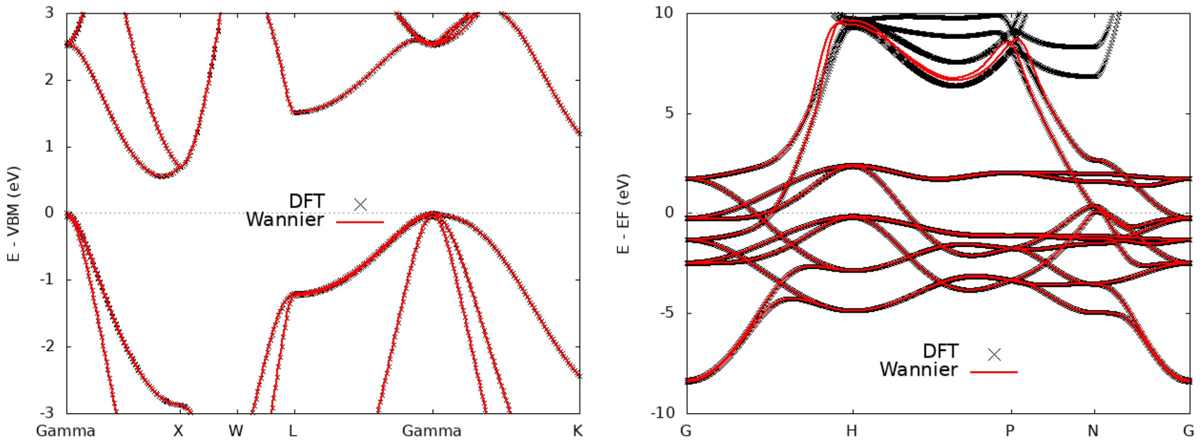

Supplementary Figure 1: Wannier-interpolated band structure of (a) silicon and (b) iron compared with that directly calculated by DFT.

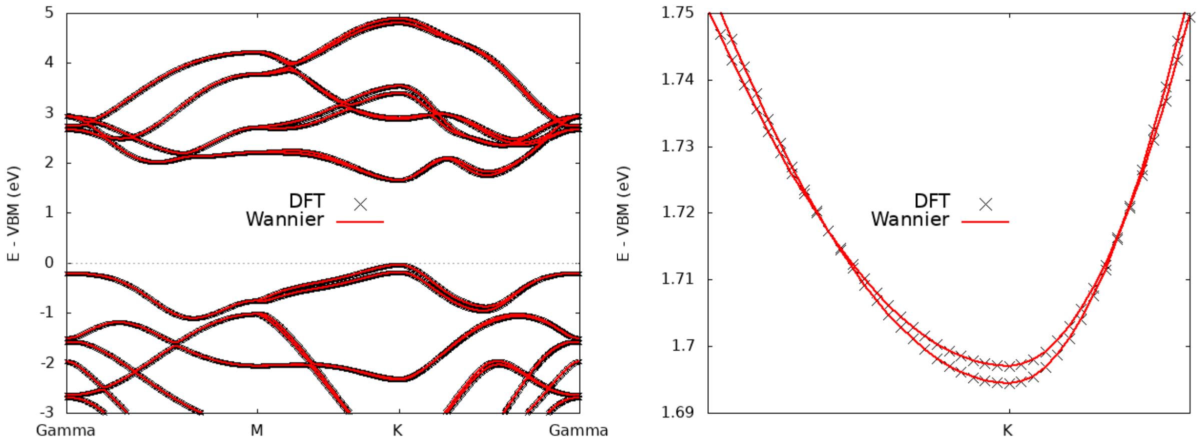

Supplementary Figure 2: Wannier-interpolated band structure of MoS_2 compared with that directly calculated by DFT in a wide energy range (left panel) and close to CBM (right panel).

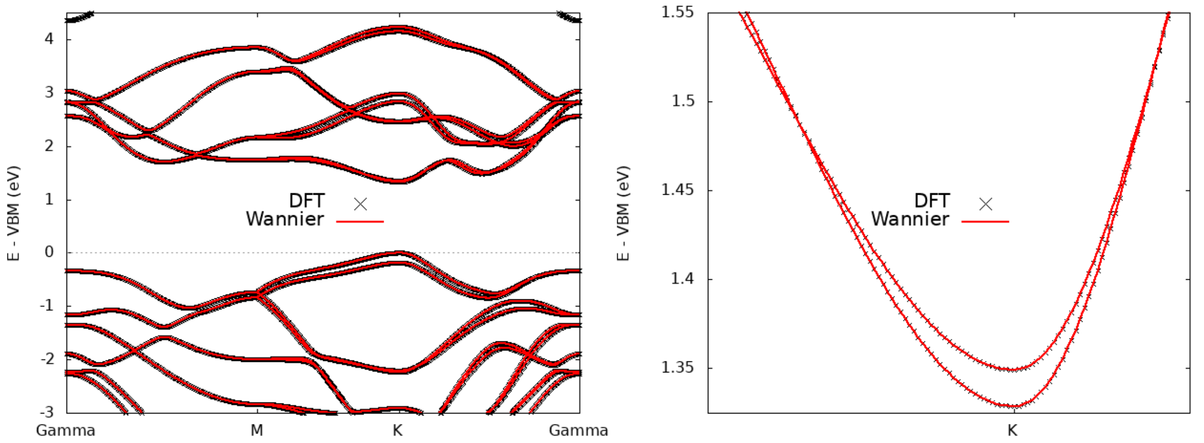

Supplementary Figure 3: Wannier-interpolated band structure of $MoSe_2$ compared with that directly calculated by DFT in a wide energy range (left panel) and close to CBM (right panel).

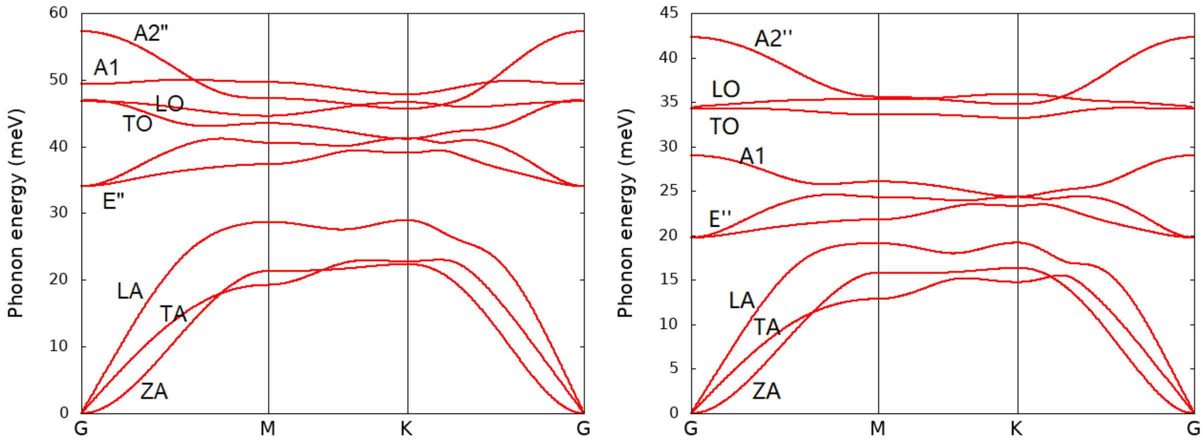

Supplementary Figure 4: Phonon dispersion of (a) monolayer MoS_2 and (b) monolayer $MoSe_2$ calculated with the supercell method using a 6×6 supercell.

Supplementary Table I: The calculated parameters of 2D Fröhlich model - in-plane (ϵ_p^m) and out-of-plane (ϵ_z^m) monolayer dielectric constants, thickness t and in-plane (Z_p^*) and out-of-plane (Z_z^*) Born effective charges, of Mo atom of MoS_2 and $MoSe_2$. See the detailed definition of ϵ_p^m, ϵ_z^m and thickness t in Ref. 3.

	ϵ_p^m	ϵ_z^m	t (bohr)	Z_p^*	Z_z^*
MoS_2	16.8	16.6	10.3	-1.0	-0.07
$MoSe_2$	18.3	18.3	11.1	-1.4	-0.08

We follow the approaches in Ref. 3 and 4 to consider the effects of the 2D Fröhlich interaction on the phonon dispersion and the electron-phonon matrix elements. The parameters of the 2D Fröhlich model are extract from a series of density functional perturbation theory[5] calculations using Quantum Espresso[6]. The model parameters are summarized in Supplementary Table I.

Supplementary Note 5: Transport properties

We show our calculated transport properties of silicon and iron compared with experimental data for verifying the implementation of our carrier lifetime due to electron-phonon scatterings. From Supplementary Figure 5, our calculated electron mobilities are in good agreement with experimental data in Ref. 7. Our calculated electrical

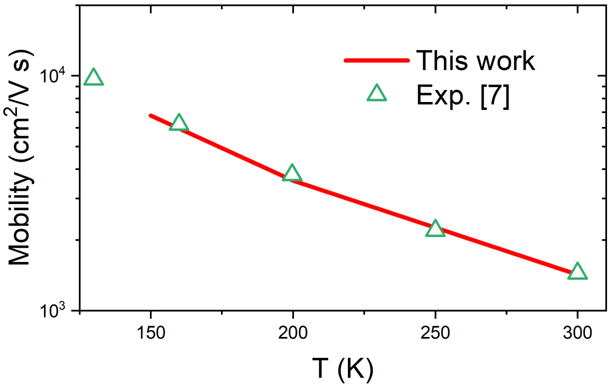

Supplementary Figure 5: Comparison between calculated and measured intrinsic (low carrier concentration $\leq 10^{15}$ cm^3) electron mobilities of silicon, as a function of temperature.

resistivity of iron at 300 K is 105 $\mu\Omega\,\mathrm{cm}$, close the experimental value 99.8 $\mu\Omega\,\mathrm{cm}$[8].

References

[1] Rosati, R., Dolcini, F. & Rossi, F. Electron-phonon coupling in metallic carbon nanotubes: Dispersionless electron propagation despite dissipation. *Phys. Rev. B* **92**, 235423 (2015).

[2] Restrepo, O. D. & Windl, W. Full first-principles theory of spin relaxation in group-iv materials. *Phys. Rev. Lett.* **109**, 166604 (2012).

[3] Sohier, T., Calandra, M. & Mauri, F. Two-dimensional fröhlich interaction in transition-metal dichalcogenide monolayers: Theoretical modeling and first-principles calculations. *Phys. Rev. B* **94**, 085415 (2016).

[4] Sohier, T., Gibertini, M., Calandra, M., Mauri, F. & Marzari, N. Breakdown of optical phonons' splitting in two-dimensional materials. *Nano Lett.* **17**, 3758–3763 (2017).

[5] Baroni, S., De Gironcoli, S., Dal Corso, A. & Giannozzi, P. Phonons and related crystal properties from density-functional perturbation theory. *Rev. Mod. Phys.* **73**, 515 (2001).

[6] Giannozzi, P. *et al.* Quantum espresso: a modular and open-source software project for quantum simulations of materials. *J. Condens. Matter Phys.* **21**, 395502 (2009).

[7] Ludwig, G. & Watters, R. Drift and conductivity mobility in silicon. *Phys. Rev.* **101**, 1699 (1956).

[8] Haynes, W. M. *CRC handbook of chemistry and physics* (CRC press, 2014).
